# Evaluation of muscle energy in isometric maintenance as an index of muscle fatigue in roller speed skating

**DOI:** 10.3389/fspor.2023.1153946

**Published:** 2023-03-23

**Authors:** Giulia Bongiorno, Helena Biancuzzi, Francesca Dal Mas, Luca Miceli

**Affiliations:** ^1^ Friuli Riabilitazione Rehabilitation Center, Roveredo in Piano (PN), Italy; ^2^ Department of Pain Medicine, IRCCS C.R.O. National Cancer Institute of Aviano, Aviano, Italy; ^3^ Department of Management, Ca’ Foscari University of Venice, Venice, Italy

**Keywords:** fatigue, surface electromyography, roller skate, muscular asymmetry, injury prevention

## Abstract

Roller speed skating is a discipline in which muscle fatigue plays an important role in athletes; in this work, we wanted to evaluate whether a methodological approach based on the energy required to maintain an isometric muscle contraction for one minute, indexed on the MVIC (maximum voluntary isometric contraction), i.e. % RMS/MVIC can give results similar to the frequency decay analysis/time in terms of usability for athlete training and injury prevention. Right and left gluteus maximus and vastus lateralis muscles (involved in the propulsive phase of skating ) were examined separately by surface electromyography in three competitive athletes in short-track speed skating on asphalt. The results showed an asymmetry between the right (less resistant) and left (more resistant) lower limb, in all three athletes, from the point of view of fatigue, in both investigated muscles. Furthermore, a trend in terms of fatigue resistance was found that was directly proportional to skill in both muscles studied. This can be of help in better planning the training of street speed skaters (which although similar to the discipline on ice is not completely superimposable) with the dual purpose of improving their performance and preventing injuries, often linked to the degree of right-left muscle asymmetry.

## Introduction

Speed roller skating is a discipline in which muscle fatigue, which is defined as the reduction of muscle capacity to perform work after previous physical effort (1, 2) plays an important role in athletes. There are few experiences in the literature in this specific area, which is different, although similar, to hockey and ice speed skating (3). In the fatigue literature dealing with surface electromyography (sEMG), there has been an emphasis on the importance of static measurement, i.e., of the muscle during isometric contraction (4). In this measurement, muscle fatigue is reflected in an amplitude increase and mean frequency decrease over time of the sEMG signal power spectrum in the tested muscle groups (5). Two recent works in particular have explored muscle fatigue and asymmetries in competitive ice skaters using this analysis methodology (6, 7). However, there are also reports that physical exertion does not affect the reduction of sEMG signal frequency (8). Referring to the experiences already published (6, 7), we wanted to verify if a methodological approach based on a simplified measure of amplitude increase of sEMG signal power during fatigue evaluation (4) (we propose the use of percentage of mean RMS - root mean square - required to maintain an isometric muscle contraction for one minute/MVIC–maximum voluntary isometric contraction) can give similar results in terms of muscle fatigue asymmetry detection. We tested this protocol on two muscles of the lower limb in three female roller-skating athletes.

## Materials and methods

Three female athletes (average height 155 cm + 2 cm DS, average weight 45 + 2 kg DS) in short-track speed skating on the road, all belonging to the same federal competitive club, were studied. All three athletes had not exercised in the previous 48 h and were in good physical shape. In the coach's opinion, the level of competitive maturity of the three athletes was in increasing order (athlete one less mature than athlete two, in turn, less mature than athlete three).

The bioelectrical activity test was conducted on the right and left gluteus maximus muscle (both important in the propulsive phase of road skating (3) and the right and left vastus lateralis, using the Freeemg 1000 sampling frequency electromyograph (BTS, en-gineering, Garbagnate Milanese, Italy). Specifically, the vastus lateralis muscle, an im-portant agonist of the propulsive movement analyzed (3), was studied to have a second comparison muscle in the study of any asymmetries, as suggested in the literature (6, 7). Before testing, the electrode attachment site was shaved and cleaned with an alcohol swab to minimize skin impedance. The bipolar pre-gelled electrodes (Ag/AgCl) had a diameter of 10 mm and the distance between the electrodes centers was 2 cm. The surface electrodes were placed on the belly of the muscle between the movement point and the tendon insertion, along the midline longitudinal axis of the muscle, according to the SENIAM methodology (9). The study protocol for each muscle evaluation required a phase of maximum isometric contraction to obtain the MVIC value and them the registration of RMS (root mean square) value during an isometric contraction at 20% of MVIC, maintained for 60 s, with the methodology described by Kaartinen et al. (10) as regards the execution of the MVIC test and already used in our previous experiences (3). The values obtained are expressed as mean %RMS/MVIC. A %RMS/MVIC value was therefore obtained for each of the four muscles investigated (right and left gluteus maximus, right and left vastus lat-eralis). The Freeemg 1000 system had the following technical specifications: device noise floor less than 1 µV RMS, input impedance greater than 100 Momh, sample rate of 1,000 Hz, reinforcement: 500. The raw EMG signals were processed into a value root mean square (RMS) with a 50 ms window. A 20–450 Hz band pass filter was used. Signal pro-cessing and EMG analysis were performed using Emg analyze software (BTS bioengineering, Garbagnate Milanese, Italy). The collected data were processed by statistical analysis with Wilcoxon test (given the smallness of the sample) with the aim to evaluate the difference between the right side and left side of the muscles investigated. The software used was SPSS (Armonk, United States). Statistical significance considered for *p* < 0.05.

## Results

**Figure 1:**
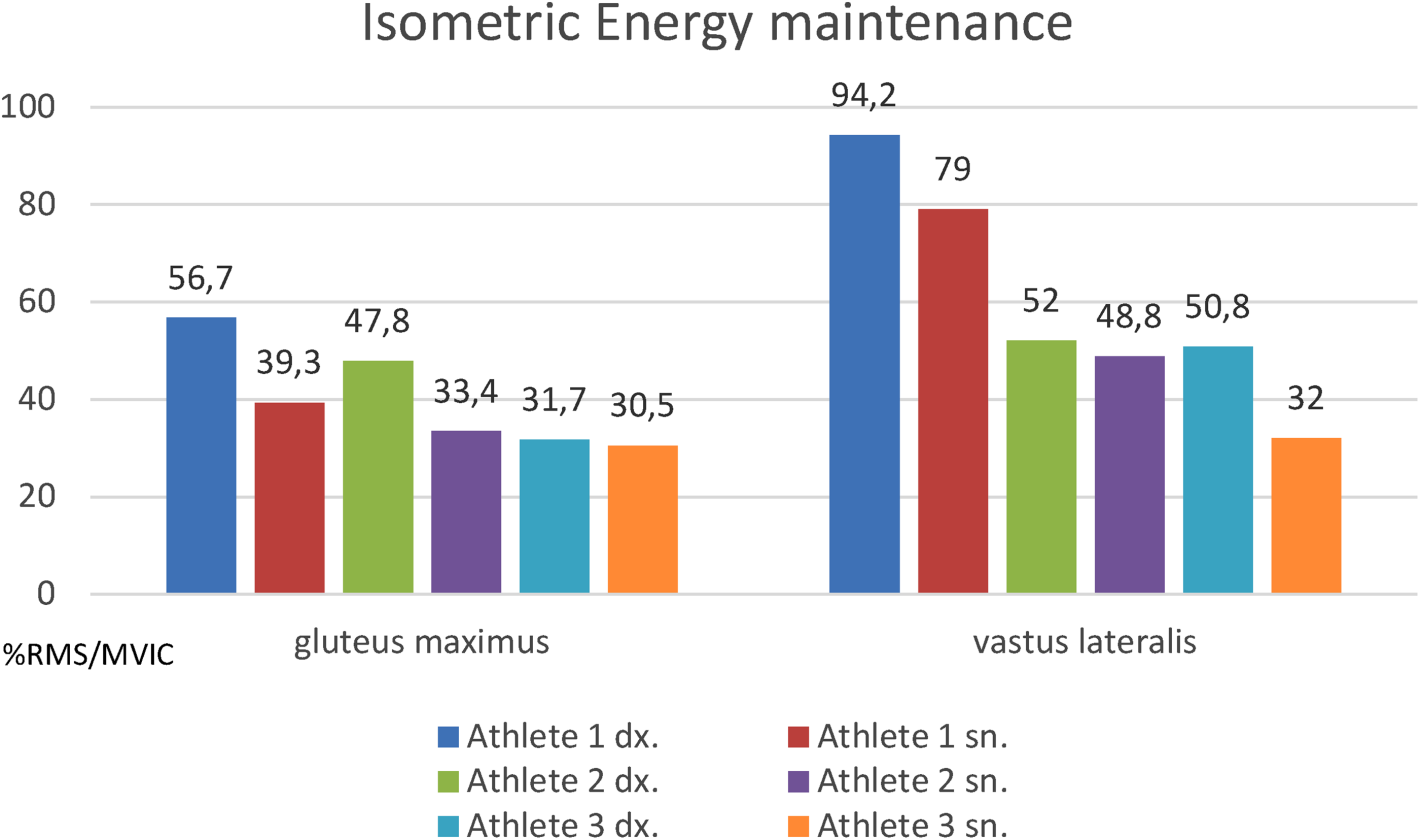
% RMS/MVIC (in ordinates, y axis) in the three athletes studied, right side and left side for the two investigated muscles. *W* = 0 with *p* < 0.05 for Wilcoxon test.

The results are indicated in Figure 1. For all three athletes there is a difference between the right side and the left side in favor of the left side (lower values of % mean RMS/MVIC) in terms of energy required to hold the 20% percent of the MVIC for 60 s, with sta-tistical significance (Wilcoxon w value of 0, with *p* < 0.05). Moreover, the trend indicates that the athlete with more competitive experience has a higher resistance to fatigue capacity than the athlete with intermediate experience and this in turn superior to the athlete with less experience. The phenomenon applies to both the gluteus maximus muscle and the vastus lateralis muscle, with the same trends, in a manner consistent with what has been observed in other works using the frequency/time decay methodology (6, 7). All three athletes also have a greater resistance to fatigue in the left muscles. compared to those on the right, also in a manner consistent with the data reported in the Literature with the frequency/time decay method (6, 7).

## Discussion

The aim of the study was to test a simplified method to what has already been published in the literature (5, 6) relating to the use of the amplitude increase in the sEMG signal during fatigue tasks to confirm the utility of a measure of asymmetry in neuromuscular fatigue; specifically, muscle energy in isometric maintenance was investigated as an index of muscle fatigue in roller speed skating. In accordance with what was suggested by other authors (5, 6), a second muscle under analysis was added (vastus lateralis muscle) in addition to the gluteus maximus muscle. A further aim of the study was to understand whether the conclusions already published on ice skaters can be extrapolated and confirmed also in short-track speed skaters on asphalt, on which the Literature is still rather scarce (3). The study of muscle asymmetries appears quite important as an object of study since in this sport athletes always turn counterclockwise, with asymmetrical movements during turns, which can expose them to important muscle asymmetries with potential implications from the point of view of injuries. Felser et al. (11), described the differences in the symmetry of the bioelectric muscle activity during skating in different sections of the ice rink: the Authors showed the occurrence of increased bioelectric activity in the right leg during curves on the ice rink and it is conceivable that this phenomenon also happens to street skaters. Noting these differences between the two sides, it can be said that the fatigue of the left and right lower limb muscles will be different.

It would be a confirmation of a generally known trend in short track, which involves a greater load, and therefore greater fatigue of the right lower limb during ice skating because of its greater load on curves (11). Since muscle asymmetries can lead to a greater probability of injury, already investigated in other sports such as football (12), the fact that this phenomenon occurred in all three athletes studied and in both muscles investigated is an important starting point for athletic trainers and rehabilitators, above all in the light of the peculiar detection of a correlation between the athlete's performance level (increasing from athlete 1 to athlete 3) and resistance to fatigue (increasing from athlete 1 to athlete 3, understood as less energy required to maintain isometric conditions for the duration of 60 s).

## Limitations and conclusions

The small number of athletes studied is a limitation of the study and will be the subject of future insights based on the preliminary data presented, although the phenomena and trends observed are superimposable to those already reported in the literature with larger cases. Furthermore, the analysis of a second muscle (vastus lateralis) in addition to the gluteus maximus muscle led to the same results, and this leads us to believe that the proposed method of fatigue analysis by %RMS/MVIC with the proposed methodology could be a useful alternative to investigate this phenomenon and the phenomenon of muscle asymmetries in athletes in a simple, rapid and non-invasive way, also in the context of short track speed skating athletes as well as in those of ice. In conclusion, the intention of the authors is to offer an objective simplified tool in the field of sEMG increase during fatigue tasks for analyzing muscle fatigue for future developments, which may range from short track roller and/or ice skating to other areas, both sports and rehabilitation.

All procedures performed on female athletes complied with the 1964 Declaration of Helsinki and its subsequent amendments.

## Data Availability

The original contributions presented in the study are included in the article/Supplementary material, further inquiries can be directed to the corresponding author.
